# Reporting the first minimally invasive spine surgery series in Nigeria: a descriptive single-center retrospective cohort study

**DOI:** 10.1371/journal.pgph.0004463

**Published:** 2025-09-24

**Authors:** Oluwafemi F. Owagbemi, Temitayo O. Ayantayo, Olawale A. R. Sulaiman

**Affiliations:** Department of Neurosurgery, RNZ Clinic, Victoria Island, Lagos State, Nigeria; PLOS: Public Library of Science, UNITED STATES OF AMERICA

## Abstract

Minimally invasive spine surgery (MISS) has gained traction since its introduction into the spine surgery armamentarium, resulting in better outcomes than the traditional open approaches. It was only recently introduced in Nigeria, where it is rarely performed. In a bid to improve access to state-of-the-art neurosurgical services in his home country, the senior author, having practiced MISS in the United States, started performing it in Nigeria in 2017. We aim to describe our MISS experience in Nigeria, a lower-middle-income country (LMIC) with high poverty indices, and report the first series of these surgeries in the country. This is a descriptive single-center retrospective cohort study performed through a review of our database of patients who had MISS for degenerative disc disease involving the thoracic, lumbar, and lumbosacral spine regions from April 2017 to May 2022. Demographic, perioperative parameter (surgery duration [SDn], estimated blood loss [EBL], length of hospital stay [LOS]), perioperative surgical site complication, and patient-reported outcome (Numeric Rating Scale [NRS] and Oswestry Disability Index [ODI]) data were retrieved and analyzed. Minimal important differences (MID) in the patient-reported outcomes (PROs) were defined as improvements of a two-point change for NRS and a 5.9–20-point difference for ODI. The same lead surgeon performed the procedures with similar operative techniques and perioperative management. The data of the 143 patients were not normally distributed. The median age was 62 years, and males comprised 55.9%. Fifty-one percent of the patients had minimally invasive (MIS) laminectomy; 45.5% and 3.5% had MIS-transforaminal lumbar interbody fusion (TLIF) and MIS-microdiscectomy, respectively. Most (73.4%) were in the lumbar spine, 25.2% involved the lumbosacral junction, and 1.4% were in the thoracic spine. Median SDn, EBL, and LOS were 112 mins, 50 mL, and 3 days, respectively. The perioperative surgical site complication rate was 4.9%. The PROs showed statistical improvement and MID between baseline and one-year follow-up. This study’s perioperative parameters and surgical site complication rates are comparable to those obtained from previous work on MIS lumbar decompression (laminectomy and microdiscectomy) and MIS-TLIF in higher-income countries. Additionally, the patients’ improvements in the PROs were comparable to findings in spine surgery research in higher-income countries. Our efforts to introduce MISS as part of our practice in Nigeria are informed by the need for deploying, developing, and maintaining beneficial cutting-edge care in LMICs where the capacity exists, while not neglecting the ‘stock’ procedures. MISS is available in Nigeria, and it is characterized in our practice by satisfactory perioperative parameters, perioperative surgical site complication rates, and PROs that are comparable with those obtained from MISS and other spine surgeries performed in countries with high incomes, where MISS is rife.

## Introduction

Low back pain (LBP) is the leading cause of disability globally, and its incidence, prevalence, and associated disability-adjusted life years have increased over the last two decades because of the rising life expectancy [1–3]. Lumbar degenerative disc disease (DDD) is a common cause of chronic LBP in adults [4], while its less common counterpart, thoracic DDD, is potentially more pernicious because of the narrower thoracic spinal canal compared with the lumbar spine and the risk of thoracic myelopathy [5]. DDD, in general, is a leading disorder necessitating care in Nigeria and globally [4,6–9].

The surgical treatment of thoracic and lumbar DDD has evolved over the years from decompression to instrumented fusion of various kinds, with minimally invasive techniques recently added to the surgical repertoire [10,11]. Minimally invasive spine surgery (MISS) has gained traction since its introduction into the spine surgery armamentarium [12]. It is designed to result in reduced collateral tissue damage and morbidity, and a faster functional recovery compared to the traditional open approaches, all with the same surgical goal [13]. True to design, the tissue damage that results from open posterior spine approaches, particularly in the multifidus muscle [14–17], is statistically lower in MISS [18]. Surgery duration, estimated blood loss, length of hospital stay, and complication rate are also reduced in comparison [19–22].

Given this improved outcome, it is no wonder that MISS is increasingly employed in many parts of the world [23,24], despite its steep learning curve [25–27] and the need for more advanced equipment than required for the open approaches [28,29]. The story appears different in some regions, however. In a global survey of spine surgeons, only half (the lowest proportion in the study) of the 16 respondents in the Africa and Middle East region thought that MISS was considered mainstream in their areas and practice settings [30], while its utilization for fusion in the region was one of the lowest in another worldwide survey [31].

MISS has been reported for the treatment of spine trauma, tuberculosis, and degenerative disease in Tanzania and South Africa (both African countries) [32–34], and has also been used in Haiti [35]. Tanzania and Haiti, like Nigeria, are classified as lower-middle-income economies, while South Africa is an upper-middle-income economy [36]. We did not find a report of MISS from Nigeria, where, based on anecdata, it is both new and rare. In the bid to improve access to state-of-the-art neurosurgical services in his home country, the senior author (OARS), having practiced MISS in the United States (U.S.), started performing it in Nigeria in 2017.

Migration of healthcare workers from developing to developed countries contributes to the depletion of human resources for health and financial resources of the “supplier/source” countries [37–39]. Nigeria, the home country of the senior author, is one of the sub-Saharan countries with the largest proportion of physician emigrants to Western countries [38,40]. Trips back home to provide medical service are one method through which U.S.-based Nigerian physicians have given back [41]. Though it started as monthly visits, the senior author’s practice in Nigeria subsequently morphed into a continuous one.

This study evaluates specific perioperative parameters, perioperative surgical site complications, and patient-reported outcomes of the senior author's MISS experience in Nigeria as primary outcomes.

## Materials and methods

### Study design

This is a retrospective review of a cohort of MISS patients in our practice.

### Setting

Nigeria had a low Human Development Index (HDI) of 0.535 and ranked 163 of 191 nations in the 2021/22 United Nations Development Programme Human Development Report [42,43]. Lagos, the state in Nigeria where our practice is located, was reported over multiple years to have the highest HDI in the country [43,44]. With an HDI of 0.681 in the 2021/22 report, which is better than the national HDI, Lagos falls into the medium human development range, but lags behind Western countries with very high development indices, and even some African countries in the high development range [42,43]. When told through another metric, the World Bank’s Human Capital Index (HCI) of 2020, which scores the country an index of 0.36 (the HCI ranges between 0 and 1), Nigeria’s story remains one of poor development [45]. The underdevelopment affects people’s purchasing power and their access to healthcare [46].

The Nigerian healthcare system lacks many of the characteristics of a strong health system, such as good health services, a well-performing health workforce, a well-functioning health information system, equitable access to essential medical products, vaccines, and technologies, a good health financing system, and leadership and governance [47]. Thus, general healthcare provision in the country is poor, and the practice of highly specialized medical fields, like neurosurgery, is poorly supported and challenging. Despite the obstacles, with multiple efforts from neurosurgical pioneers and their progeny in Nigeria over the years, progress in the delivery of neurosurgical services has been recorded [48–56].

Our practice, RNZ Clinic, Victoria Island, Lagos, Nigeria, is private, and the surgeries were performed in a private hospital (Euracare Multi-Specialist Hospital, Victoria Island, Lagos, Nigeria) with a single operating room. A Jackson table, a Wilson frame, an operating microscope, a power drill, electrocautery, and fluoroscopy were used as standard items for MISS. Retraction was maintained during the MISS procedures using a 22 mm tubular retractor (METRx System, Medtronic, Memphis, TN, USA), while pedicle screw instrumentation was performed percutaneously. Some of the other neurosurgical conditions treated in the practice include brain and spine tumors, chronic subdural hematomas, brain and spine trauma, and peripheral nerve injuries and entrapments.

### Participants

The study participants were the patients who underwent minimally invasive spine procedures for DDD involving the thoracic, lumbar, and lumbosacral spine regions from April 2017 to May 2022 in our practice. We made the diagnosis of thoracic, lumbar, and lumbosacral DDD from a combination of clinical features of mechanical back pain, radiculopathy, and/or neurogenic claudication, with magnetic resonance imaging findings of spondylosis, spondylolisthesis, spinal canal stenosis, and/or neural foraminal stenosis. Following six weeks of unsuccessful non-operative treatment (oral medication and physical therapy, with or without an epidural steroid injection), we recommended surgery. MISS was our first choice for patients with one- and two-level disease, to whom we explained the benefits of MISS over the open approaches, allowing them to make an informed decision. Open surgery was reserved for surgery at three levels and above, and for one- and two-level revisions, where we deemed that MISS would be hazardous or unnecessarily difficult.

The senior author performed all the minimally invasive (MIS) procedures—minimally invasive transforaminal lumbar interbody fusion (MIS-TLIF), minimally invasive laminectomy (MIS-laminectomy), and minimally invasive microdiscectomy (MIS-microdiscectomy)—using a similar technique to that employed in a previous publication [19], assisted in some by the co-authors. MIS-laminectomy and MIS-microdiscectomy were grouped into minimally invasive decompression (MIS-decompression) for ease of analysis. We irrigated all wounds copiously and closed them in layers. All the patients had similar postoperative care using standardized postoperative order sets, and the care for each procedure type was nearly identical for the patients who underwent it. Patients were sent for physical therapy after surgery as needed, and they returned to work as soon as they could. The postoperative management is also similar to the senior author’s method in the U.S [19].

### Variables

The collected data include patient demographics (age in years and sex), diagnoses, and perioperative parameters, including the surgeries performed, the number of spinal levels (NOL) operated on, surgery duration (SDn), estimated blood loss (EBL), length of hospital stay (LOS), perioperative surgical site complications, patient-reported outcomes (PROs), and discharge destination. The primary outcomes are the perioperative parameters (SDn, EBL, and LOS), perioperative surgical site complications, and PROs.

SDn and EBL were determined from operating room records and LOS from admission records. SDn was defined in our cohort as the duration, in minutes (min), from the skin incision to the final closure stitch, as described by other workers [57] and recorded by the operating room staff. EBL was the blood loss, in milliliters (mL), at surgery based on visual estimation. [58,59] LOS was the duration, in days, from surgery to discharge from the hospital.

Perioperative surgical site complications identified by the surgeon were recorded in the database. They are dura tear, pseudomeningocele, early surgical site infection (SSI), malpositioned implants, implant migration, and nerve injury. A dura tear was recorded when observed during surgery or if postoperative cerebrospinal fluid (CSF) leakage was observed. A pseudomeningocele was recorded if the patient had a fluctuant swelling prompting a postoperative MRI that showed an abnormal collection of CSF outside the dura mater. We evaluated the surgical wounds per practice protocol (as needed during admission, on the seventh postoperative day, and one month after surgery, with instructions for features of wound infection given to the patient and caregivers at discharge). Early SSI was assessed based on the Centers for Disease Control and Prevention, USA criteria, and occurring within 30 days of surgery [60]. Malpositioned and migrated implants were diagnosed using postoperative computed tomography confirmation with or without dermatomal pain. Nerve injury was recorded when observed during the surgery and when the patient developed new weakness after surgery. Surgical site complication review in this study was limited to those occurring within 30 days of surgery, which speaks mostly to surgical technique and the operative environment.

The PROs that we evaluated were low back pain and leg pain, using the 11-point (from 0 for no pain to 10 for the worst imaginable pain) Numeric Rating Scale (NRS) [61], and disability, using the Oswestry Disability Index (ODI), which assesses disability on a scale of 0% to 100% [62,63]. These were assessed preoperatively and at one, six, and 24 months after surgery, using questionnaires. Minimal important differences (MID) in PROs were defined as improvements of a two-point change for NRS and a 5.9–20-point difference for ODI [64].

Apart from perioperative surgical site complications and the PROs, observation of the variables, because of their nature, was limited to the admission period. Age, SDn, EBL, and LOS are continuous variables, while NOL and PROs are ordinal discrete variables analyzed as continuous variables. Also treated as continuous variables for statistical analysis were perioperative surgical site complications and discharge, which are discrete events. Sex is categorical. The patients’ demographics, DDD data, and surgery types were the independent variables, while SDn, EBL, LOS, perioperative surgical site complication rates, and PROs constituted the dependent variables.

### Data sources and measurement

The database was accessed for the research on January 31, 2024, and only one author, one of the physicians in the practice, had access to information that could potentially identify individual participants during data collection. Patient consent was obtained for all procedures, and the Nigerian Institute of Medical Research’s Institutional Review Board granted ethical approval for the study with a waiver of informed consent.

### Statistical methods and study reporting

The data were evaluated using the Shapiro-Wilk test for normality to determine whether to use parametric or nonparametric tests, and described using counts, percentages, and medians. Non-parametric repeated measures analysis of variance (Friedman test) was used to compare the baseline PROs with the follow-up values in a pairwise fashion. We set statistical significance at p < 0.05 [65,66], and missing data were addressed using the available-case analysis [67–69]. We performed the statistical analysis with *jamovi* (The jamovi project, Sydney, Australia) [70] and implemented the Strengthening the Reporting of Observational Studies in Epidemiology (STROBE) guideline in reporting this work [71].

## Results

A total of 261 patients had spine surgery during the 62-month study period, 143 of whom had minimally invasive spine procedures for DDD involving the thoracic, lumbar, and sacral spine regions. This constitutes 54.8% of our spine surgery practice and 65.0% of the 220 patients operated on for DDD during the period. The excluded patients had open spine surgeries and/or surgery for non-degenerative spine conditions. When tested for normality, the data were not normally distributed.

**Fig 1:**
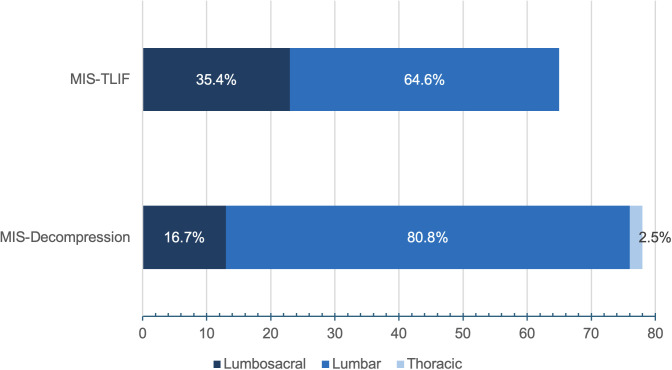
Spinal regions operated on—minimally invasive transforaminal interbody fusion (MIS-TLIF) and minimally invasive decompression (MIS-decompression), which comprises minimally invasive laminectomy and minimally invasive microdiscectomy.

The median age was 62 years, with an interquartile range (IQR) of 55–70 years, and males comprised 55.9% (80/143). More than half (73, 51.0%) of the patients had MIS-laminectomy, while 65 (45.5%) and five (3.5%) patients had MIS-TLIF and MIS-microdiscectomy, respectively. Overall, 105 (73.4%) of the surgeries were performed in the lumbar spine, while 36 (25.2%) involved the lumbosacral junction, and the remaining two were in the thoracic spine (Fig 1). All the MISS were performed in one (82, 57.3%) or two (61, 42.7%) spinal levels.

The median (IQR) SDn, EBL, and LOS were 112 (64–185) min, 50 (30–100) mL, and 3 (3–3) days, respectively. There were three dura tears (2.1%), two incidents of malpositioned screws (1.4%), and one SSI and nerve injury each (0.7% each), with a total complication rate of 4.9%. A subanalysis of the patients into MIS-TLIF and the MIS-decompression is shown in Table 1.

**Table 1:** Description of patient characteristics, perioperative parameters, and perioperative surgical site complications, with subdivision into minimally invasive transforaminal interbody fusion and minimally invasive decompression, which comprises minimally invasive laminectomy and minimally invasive microdiscectomy.

Variable		Total	MIS-TLIF*	MIS-Decompression^†^	Missing Data, n
Number of cases		143	65	78	0
Sex n (%)	Male	80 (55.9)	28 (43.1)	52 (66.7)	0
	Female	63 (44.1)	37 (56.9)	26 (33.3)	
Median (IQR) age, years		62 (55–70)	61(53–68)	63 (56–71)	0
Number of levels, n (%)	1-level	82 (57.3)	40 (61.5)	42 (53.8)	0
	2-level	61 (42.7)	25 (38.5)	36 (46.2)	
Median (IQR) SDn, min		112 (64–185)	190 (145–219)	69 (93–53)	2*; 3^†^
Median (IQR) EBL, mL		50 (30–100)	100 (50–100)	30 (30–50)	3*; 3^†^
Median (IQR) LOS, days		3 (3–3)	3 (3–3)	3 (2–3)	1^†^
Perioperative surgical site complication rate, n (%)		7 (4.9)	6 (9.2)	1 (1.3)	0
Discharge n (%)	Inpatient PT	76 (59.8)	46 (73.0)	30 (46.9)	2*; 14^†^
	Home (no skilled care)	50 (39.4)	16 (25.4)	34 (53.1)	
	Died	1 (.8)	1 (1.6)	0 (.0)	

MIS-TLIF: Minimally invasive transforaminal lumbar interbody fusion.

MIS-Decompression: Minimally invasive decompression.

IQR: Interquartile range.

SDn: Surgery duration.

EBL: Estimated blood loss.

LOS: Length of hospital stay.

PT: Physical therapy.

Annotations (* and ^†^) indicate the group in which the annotated missing data occurs.

Each malpositioned screw required an additional procedure for revision in the two affected patients. The patient who developed SSI had undergone MIS-TLIF and was one of those who had a dura tear, which led to postoperative CSF leakage, for which he had a second procedure for dura repair. The SSI was a deep one, which caused prolonged admission and required an additional procedure for wound debridement and washout. The nerve injury, with an associated dura tear, occurred intraoperatively during MIS-TLIF, and this resulted in a right-sided foot drop, which improved with physiotherapy. The third dura tear occurred during an MIS-laminectomy, being the only perioperative surgical site complication in that group. The latter two dura tears were repaired in the same surgery with no postoperative CSF leakage. There was one mortality—a morbidly obese female who died on the seventh postoperative day after developing sudden severe pulmonary embolism symptoms and rapid clinical deterioration. There were no perioperative surgical site complications in the patients who had MIS-microdiscectomy.

**Fig 2:**
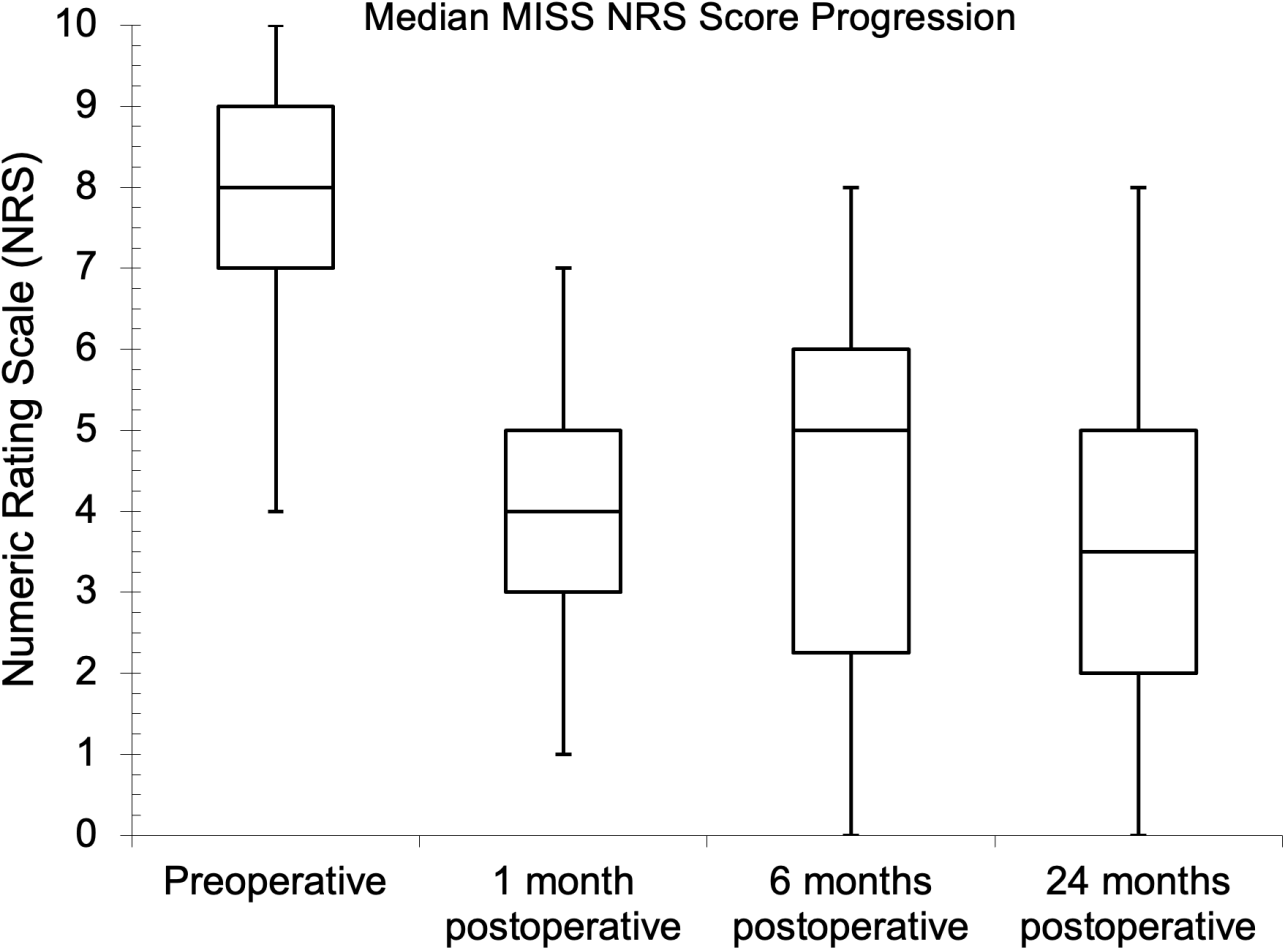
Median minimally invasive spine surgery (MISS) Numeric Rating Scale scores at preoperative and follow-up evaluations.

**Fig 3:**
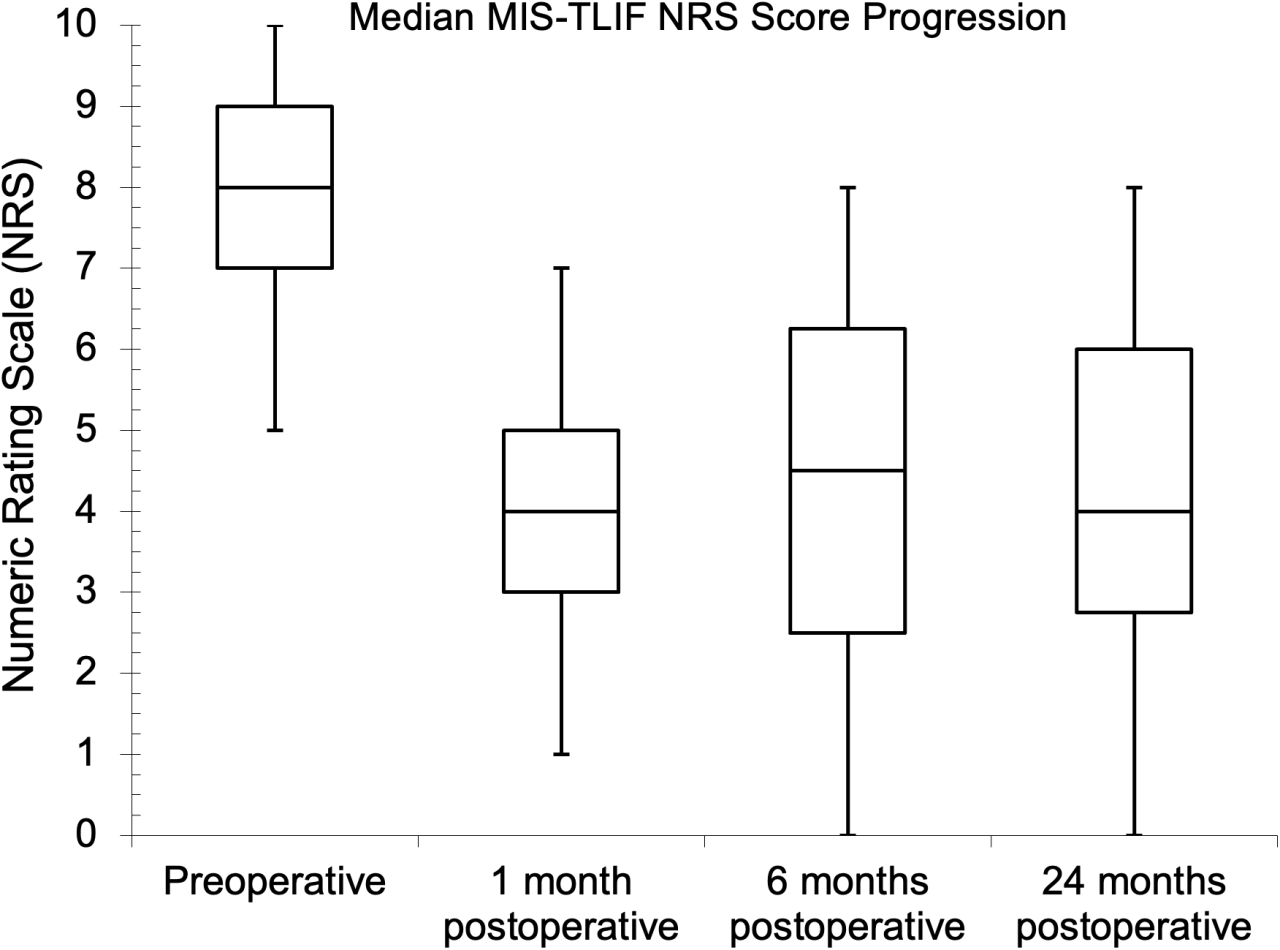
Median minimally invasive transforaminal lumbar interbody fusion (MIS-TLIF) Numeric Rating Scale scores at preoperative and follow-up evaluations.

**Fig 4:**
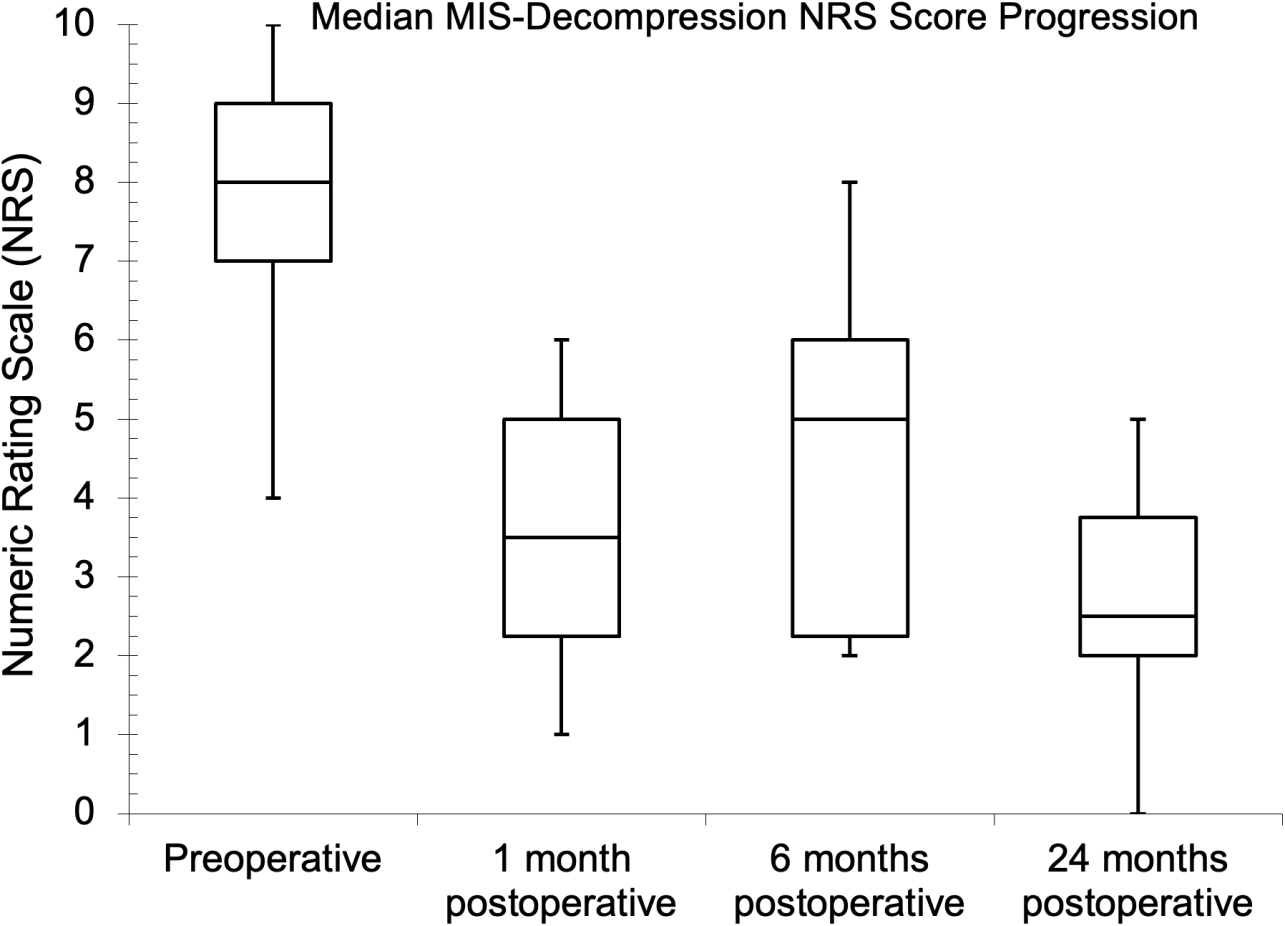
Median minimally invasive decompression (MIS-decompression) Numeric Rating Scale scores at preoperative and follow-up evaluations.

**Fig 5:**
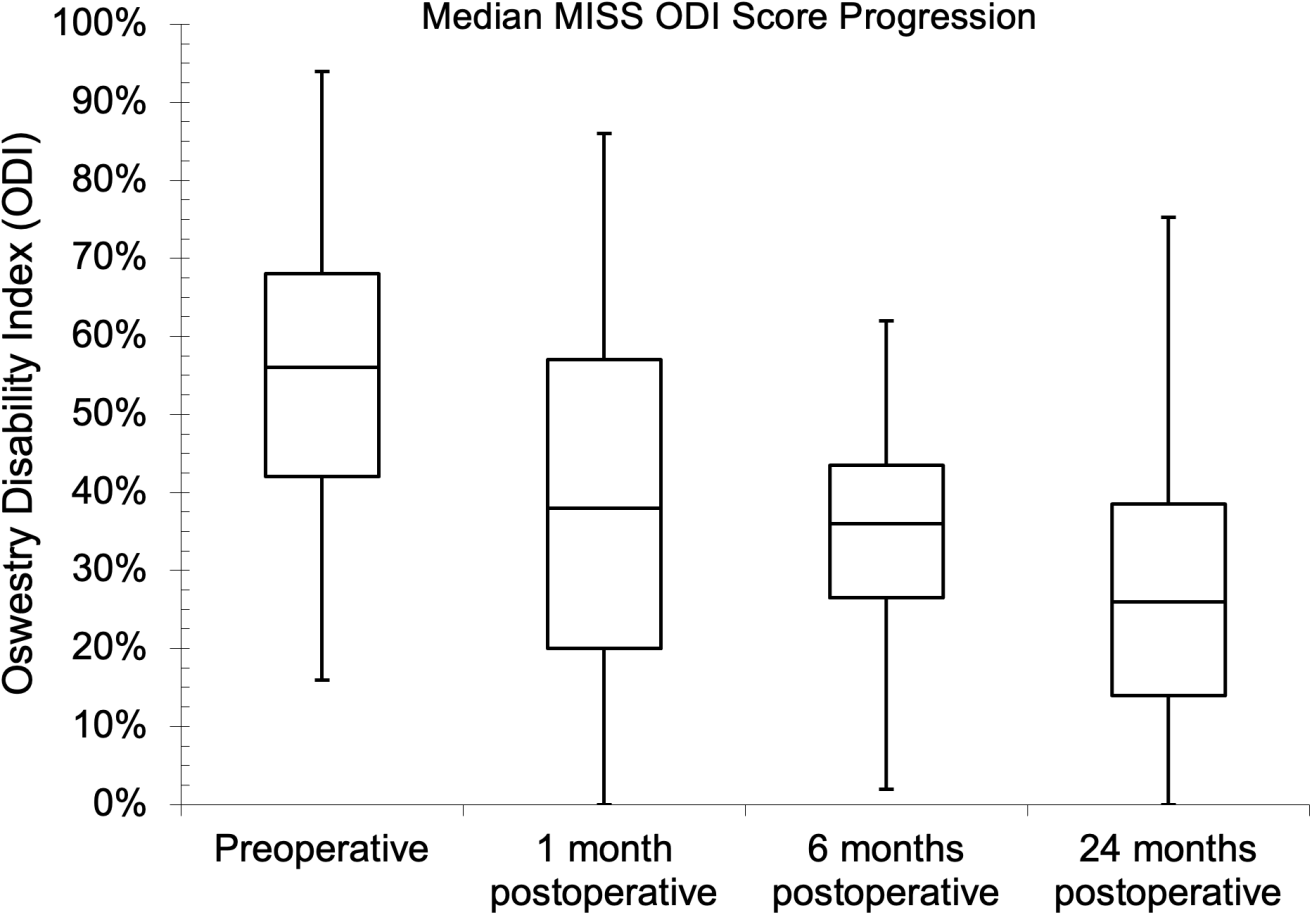
Median minimally invasive spine surgery (MISS) Oswestry Disability Index scores at preoperative and follow-up evaluations.

**Fig 6:**
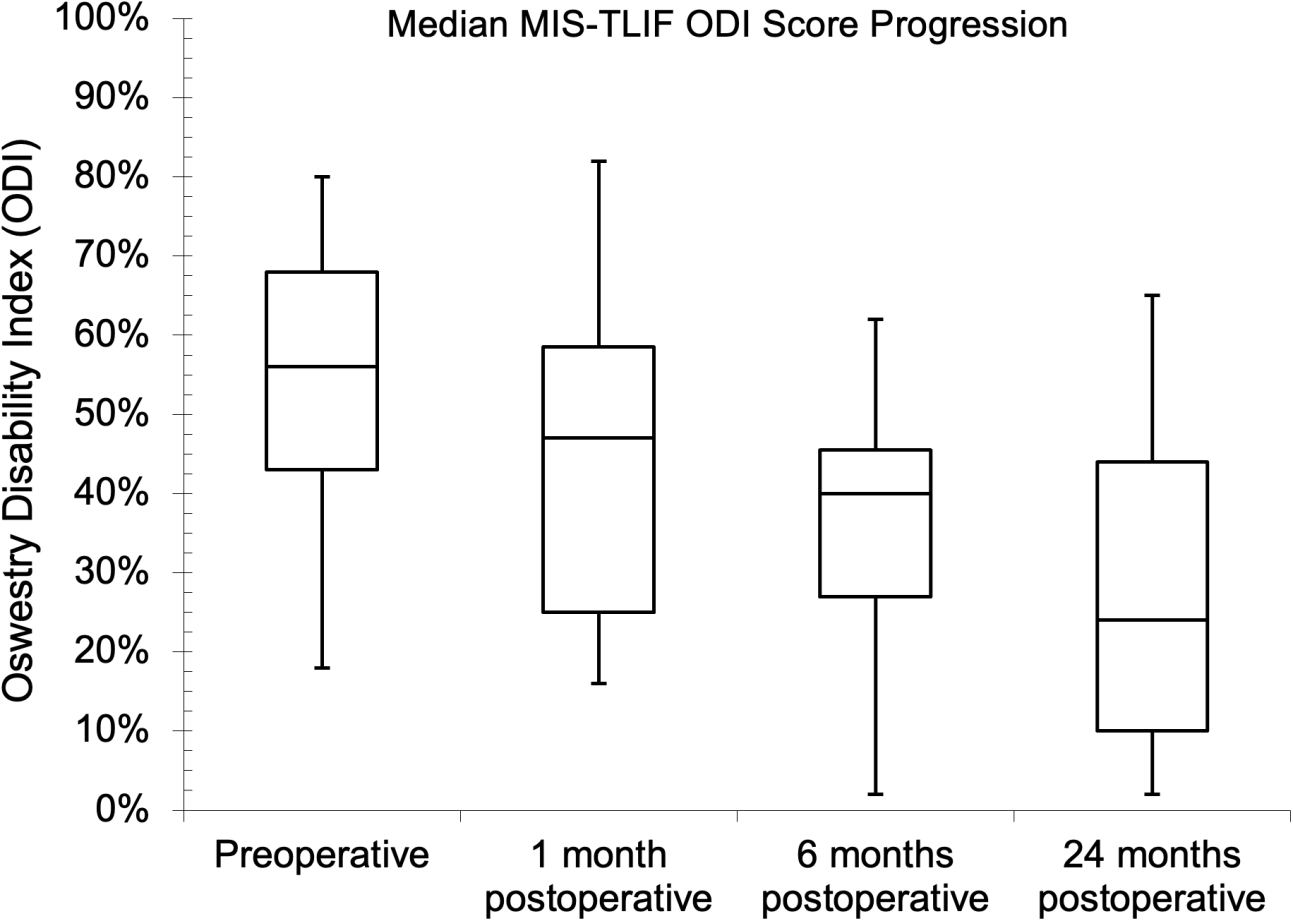
Median minimally invasive transforaminal lumbar interbody fusion (MIS-TLIF) Oswestry Disability Index scores at preoperative and follow-up evaluations.

**Fig 7:**
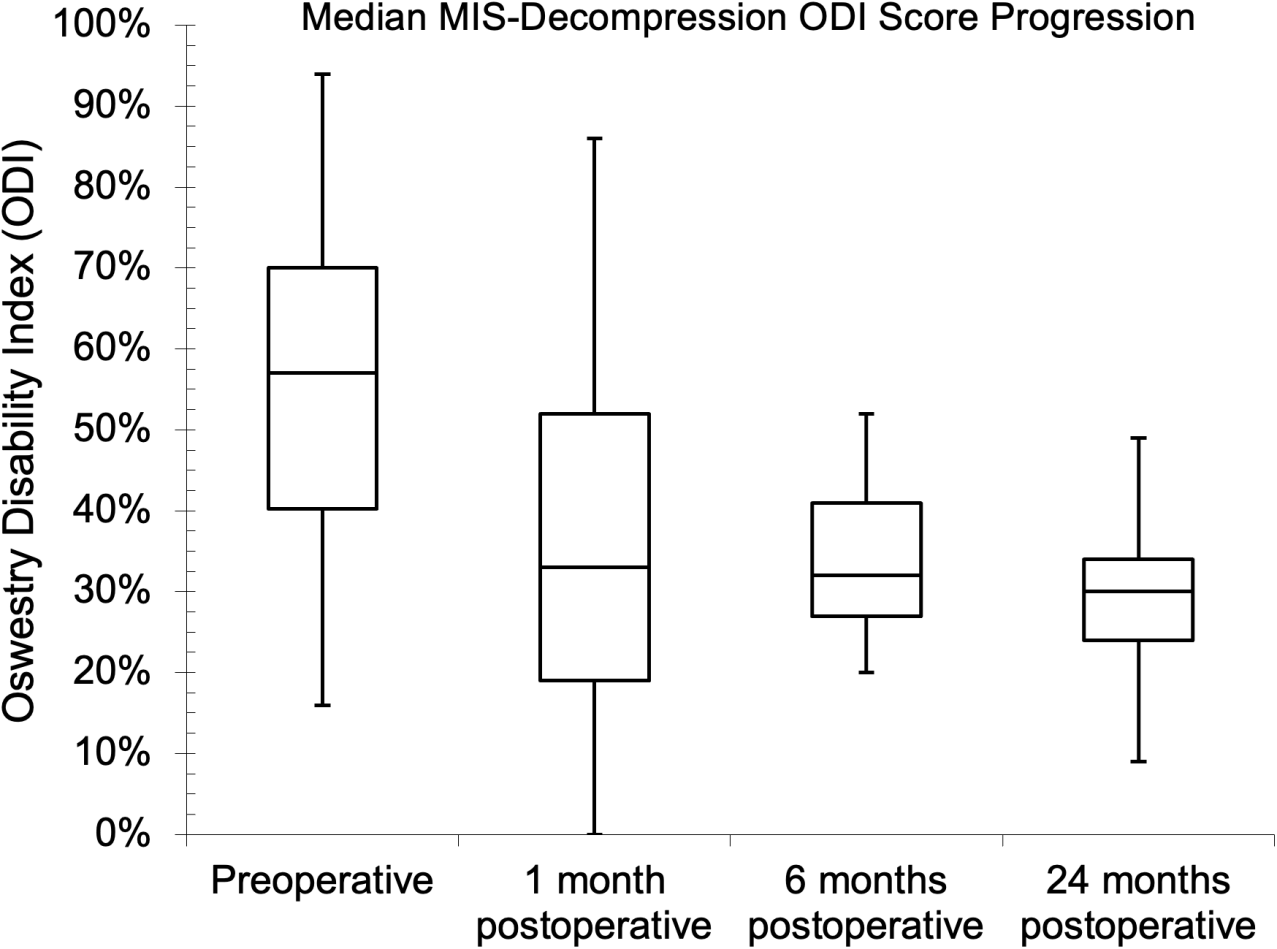
Median minimally invasive decompression (MIS-decompression) Oswestry Disability Index scores at preoperative and follow-up evaluations.

Seventy-five and 81 of the patients had preoperative NRS and ODI assessments, respectively, while the follow-up assessment rates were 45.3% and 43.2% at one month for NRS and ODI, respectively. The follow-up assessment rates were 29.3% for NRS and 32.1% for ODI at both six and 24 months. The median (IQR) baseline NRS score was 8.0 (7.0–9.0). Postoperatively, the median (IQR) scores were 4.0 (3.0–5.0), 5.0 (2.3–6.0), and 3.5 (2.0–5.0) at one, six, and 24 months, respectively. There was a statistical difference between the baseline NRS score and the six- and 24-month follow-up values (p = 0.041 and p = 0.023, respectively) during pair-wise comparison. The preoperative median (IQR) ODI score was 56.0% (42.0–68.0%), while it was 38.0% (20.0–57.0%), 36.0% (26.5–43.5%), and 26.0% (14.0–38.5%), respectively, one, six, and 24 months after surgery. There was also a statistical difference between the baseline ODI score and the follow-up values (p = 0.041, p = 0.004, and p < 0.002, respectively) during pairwise comparison. The PROs showed improvements with MID between baseline and one-year follow-up. The NRS and ODI results are further illustrated in Figs 2–7.

Most (76, 59.8%) of the patients were discharged to inpatient physical therapy, while 39.4% (50) were discharged home with no skilled care. A statistically higher proportion of patients less than 65 years of age were discharged home compared to patients greater than/equal to 65 years of age (p = 0.01).

## Discussion

The results of this study describe the patients, indications, practice, perioperative parameters, perioperative surgical site complications, and patient-reported outcomes of MISS performed by the authors in Nigeria. Though the data were prospectively collected, some were missing. They were assumed to be missing completely at random (MCAR) since they were not observed to be related to other variables [67–69]. These missing data are due to omissions in records, and we believe that applying the available-case analysis (pairwise deletion) helped keep the data as robust as possible. This approach utilizes only the data that is available for the analysis of each variable, while keeping the total sample unchanged; thus, the sample sizes differ between variables. It ensures that available data from participants with incomplete data is not lost due to the complete exclusion of the affected participants from the analysis, as would occur in a complete case analysis (listwise deletion), which would lead to a lower statistical power. Another approach, the inputational analysis, would require additional subanalysis to replace missing values [67,69].

This study’s perioperative parameters and surgical site complication rates are comparable to those obtained from previous work on MIS lumbar decompression and MIS-TLIF [19,72–83]. While there is much similarity in surgical techniques and outcome evaluation between these reports and our work, there are a few differences we wish to highlight. In contrast to this study and the work of Sulaiman et al. [19], where bilateral percutaneous screw fixation was performed, Nandyala et al. [73] used a unilateral approach, which may explain the lower SDn and EBL they found, particularly in the second arm of their study. Another difference is the much higher complication rates of Ahn et al. [72] and Nandyala et al. [73] This is likely due to their inclusion of longer-term complications, like pseudoarthrosis, which we did not set out to evaluate.

Patient-reported outcomes are important, and practitioners increasingly use them in spine surgery, particularly regarding MID, the smallest change perceived by patients as clinically important [64,84]. In our study, the patients’ improvements in PROs, which were statistically significant, were comparable to findings in spine surgery research in higher-income countries.

Patients’ home discharge rates vary across studies, given that the patient populations and techniques in these studies differ [79–83]. Our patient disposition data compare well with those from a “spine naïve” community hospital in the U.S., also reporting initial cases [80]. The dearth of skilled home care in our environment markedly hampered our ability to discharge patients needing skilled care to their homes. Our finding that the discharge to inpatient physical therapy rate was statistically higher for older patients compares with the work of Altshuler et al. [83], though they used 60 years as their threshold. Most of the patients with missing disposition data in our study were less than 65 years of age. A mock analysis of a scenario where all the patients below 65 years of age who had missing data were discharged home still yielded a statistically higher inpatient physical therapy discharge rate for older patients.

Improved access to safe and affordable surgical care is both a major need in lower-middle-income countries (LMIC) and a 2030 Global Surgery target [85]. The healthcare disparities between developed and developing nations that affect neurosurgery [86] will also need to be addressed if this need/target must be met. The neurosurgical workforce is an area of disparity, with few neurosurgeons dealing with huge loads of essential consultations and surgical cases in these developing countries, and the workforce need is projected to worsen towards 2030 [50,86]. While capacity-building efforts like twinning and training are helpful, these methods face challenges [87], some of which are likely to be mitigated by the continuous presence of the capacity builders in the countries of need, as is the case in our practice. The benefit is seen in neurosurgical care generally, but where the capacity for beneficial cutting-edge care can be deployed, developed, and maintained in low-income countries (LICs) and LMICs, we believe it is expedient to offer such care to the people in these nations, while not neglecting the ‘stock’ procedures. Moreover, while spine surgery is expensive, and MISS is more technology-driven than open spine surgeries, the total cost of patient care for undergoing MISS is generally lower than for the open counterparts, mostly related to decreased length of hospital stay, operative time, perioperative morbidity, and narcotic pain medication use. This informed our efforts to introduce MISS as part of our practice in Nigeria.

Despite the advantages of MISS (reduced complication profile, increased safety, and lower direct costs compared to open approaches), its resource-intensiveness in terms of surgical equipment has made it generally prohibitive in developing countries, leaving its availability there to the episodic interventions of practitioners from countries in the Global North [32,35,88]. The pitfalls of this approach, despite the good intentions, possibilities, and successes, have been described [88]. The feasibility of MISS as a continuous practice in the Global South rather than as isolated occurrences [32–35] is shown in this study. Though it started as monthly visits, the senior author’s MISS practice in Nigeria has morphed into a steady practice, with patient numbers approaching those of his previous practice in the U.S. If there are reports of more consistent MISS practice in the region, we have yet to find such, despite the reported acceptance and utilization in the Africa and Middle East region in the cited global surveys [30,31]. Since both ‘subregions’ were combined in the surveys, perhaps the acceptance and utilization were recorded more in the Middle East than in Africa.

With a population of about 220 million people [89], Nigeria has 40.1% of its population living below the national poverty line and 39.1% living below the international poverty line of $1.90 a day [42]. This is further worsened by poor healthcare access that is connected to poor social health insurance coverage, which was recently reported to be less than 5% of the population, with most people paying out-of-pocket for healthcare [90]. This out-of-pocket health expenditure contributes to impoverishment in the country [46]. We did not collect data on the source of funds for the surgeries, vis-à-vis health insurance versus out-of-pocket expenditure, though, anecdotally, we had patients with these two sources of funding for their surgeries.

On the backdrop of this socioeconomic scenario, and even with neurological diseases and injuries being major causes of death and disability globally [91,92], neurosurgery suffers from healthcare disparities between developed and developing countries, with LICs and lower-middle-income countries (including Nigeria) having poor access to neurosurgical care [86]. Degenerative spine disease is one of the leading conditions requiring such care globally, including in Nigeria [4,6–9]. Despite the stark realities of healthcare in Nigeria, efforts to provide safe and affordable neurosurgical care in the country are being made, especially by individuals and through private institutions, including the senior author, who moved his practice completely from the U.S. to Nigeria in 2019 [93].

It is important to strengthen health systems in source countries to reduce brain drain and its impact. Notable efforts, such as increased worker incentives and investment in healthcare systems, do not appear to have sufficiently stemmed the tide universally [38,40]. Nigeria, where a National Policy on Health Workforce Migration was recently enacted, is not left out of these efforts [94]. Other strategies that have been proposed for combating brain drain include task shifting and health exchange programs involving resources and staff, and their pros and cons have been highlighted [95]. Since brain drain involves emigration from the source countries, and is thus long-term, rather than episodic, its reversal, brain gain, cannot be limited to episodic visits, such as medical missions, if it must be successful [96,97]. The role of diaspora physicians in sustainable brain gain through long-term return to their home countries is shown in this work.

The senior author has been involved in the education and training of neurosurgery and orthopedic residents and faculty in Nigeria and has organized many training programs in both Nigeria and the USA for the same group. He facilitated spine surgical mission work in Nigeria as well. His decision to establish RNZ Clinic was driven by the high demand for spine care by Nigerian patients who traveled to the USA for this, so he decided to bring the much-needed MIS spine care back home. The critical steps included a significant investment in the education and training of the theater, clinic, and hospital medical staff, including taking some of them to the USA, investments in expensive but essential theater equipment such as the Jackson table, operating microscope, power drill, and electrocautery, as well as spine implants. He instituted protocols and standard operating procedures to standardize patient care experience and quality of care.

Our study has some limitations, including its retrospective nature, with its inherent challenges and missing data, as discussed. Additionally, there is a possibility of under-reporting perioperative surgical site complications, as some of their evaluations using imaging were guided by clinical findings. This would especially affect lower grades of the complications, which are more likely to be subclinical. Furthermore, it is a single-surgeon and single-center experience, which is not implausible since MISS is just burgeoning in Nigeria. Nonetheless, we believe it is important to highlight this work to explore the possibilities of MISS in the country, provide some guidance on starting such work in LMICs, and document an MISS series that can serve as a background for future studies in Nigeria. As the practice of MISS spreads in Nigeria, multicenter studies may corroborate our findings, making our results generalizable. One of our study’s strengths is that the patient numbers approach those of multiple studies from the developed world over similar periods. There is also strength in the evaluation of the parameters using sources free of surgeon bias, such as anesthesia and nursing records, as well as patient-filled questionnaires.

## Conclusions

MISS is available in Nigeria, and it is characterized in our practice by satisfactory perioperative parameters, perioperative surgical site complication rates, and patient-reported outcomes that are comparable to those obtained from MISS and other spine surgeries performed in countries with higher incomes than Nigeria, where MISS is a staple of spine surgery practice. This underscores the role that diaspora medical experts can play in the development of modern surgical care in Sub-Saharan African countries. A reversal of the “brain drain” to “brain gain” is slowly happening in many medical fields in Nigeria, whereby U.S.- and U.K.-trained specialists are returning to Nigeria to provide state-of-the-art healthcare to Nigerian patients.
